# LINE-1 ribonucleoprotein condensates bind DNA to enable nuclear entry during mitosis

**DOI:** 10.1126/sciadv.adt9318

**Published:** 2025-05-02

**Authors:** Sarah Zernia, Farida Ettefa, Srinjoy Sil, Cas Koeman, Joëlle Deplazes-Lauber, Marvin Freitag, Liam J. Holt, Johannes Stigler

**Affiliations:** ^1^ Gene Center Munich, Ludwig-Maximilians-Universität München, Munich, Germany.; ^2^ New York University Grossmann School of Medicine, New York, NY, USA.; ^3^ Institute for System Genetics, New York, NY, USA.

## Abstract

Long interspersed nuclear element–1 (LINE-1) is an autonomous retrotransposon that makes up a substantial portion of the human genome, contributing to genetic diversity and genome evolution. LINE-1 encodes two proteins, ORF1p and ORF2p, both essential for successful retrotransposition. ORF2p has endonuclease and reverse transcription activity, while ORF1p binds RNA. Many copies of ORF1p assemble onto the LINE-1 RNA to form a ribonucleoprotein (RNP) condensate. However, the function of these condensates in the LINE-1 life cycle remains unclear. Using reconstitution assays on DNA curtains, we show that L1 RNP condensates gain DNA binding activity only when RNA is super-saturated with ORF1p. In cells, L1 RNP condensates bind to chromosomes during mitosis. Mutational analysis reveals that DNA binding is crucial for nuclear entry and LINE-1 retrotransposition activity. Thus, a key function of ORF1p is to form an RNP condensate that gains access to the genome through DNA binding upon nuclear envelope breakdown.

## Teaser

ORF1p forms condensates that bind to chromosomes during cell division, allowing L1 elements to insert themselves into the genome.

## INTRODUCTION

Transposable elements are genetic parasites that can move within the genome (*1*). The long interspersed nuclear element–1 (LINE-1, L1) is the only autonomously active retrotransposon (*2*) in humans and accounts for about 17% of the human genome (*3*, *4*). The amplification of L1 in the human genome is one of the main drivers for genetic diversity and human evolution (*5*–*7*), but insertion into essential genes can also have severe effects on the host organism, contributing to the development of diseases such as neurodegeneration and cancer (*8*, *9*). Thus, it is of great interest to understand the mechanisms underlying retrotransposition.

**Fig. 1.**
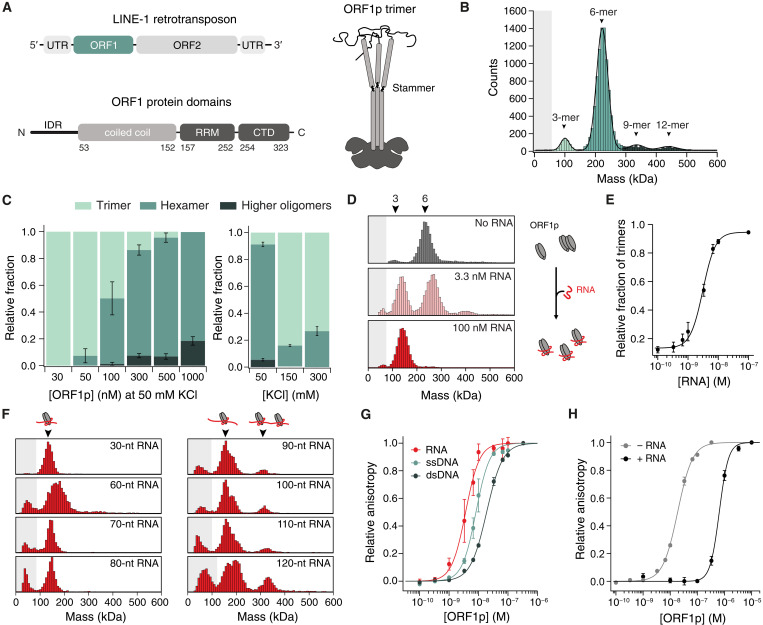
ORF1p oligomerization is influenced by RNA. (**A**) Top: Schematic representation of L1 retrotransposon coding for ORF1p and ORF2p. Bottom: Domain composition of ORF1 protein containing an N-terminal IDR, a CC followed by an RRM and a CTD. Right: Schematic representation of the ORF1p homotrimer. (**B**) Mass photometry of 300 nM ORF1p at 50 mM KCl. The hexamer is the dominant species. Gray shading illustrates the detection limit. *N* = 4. (**C**) Left: Mass photometry of ORF1p concentration series at 50 mM KCl. With higher protein concentrations, more and larger oligomers are formed. Right: Mass photometry of KCl series at 300 nM ORF1p. Oligomerization is reduced at higher salt concentrations. Means ± SEM of *N* = 3. (**D**) Mass photometry of 300 nM ORF1p mixed with increasing concentrations of 30-nt RNA. RNA presence leads to a dissociation of the hexamer and RNP formation. *N* ≥ 4. (**E**) Relative fraction of trimers at increasing RNA concentrations fitted with a Hill equation. *h* = 2.1 ± 0.3, means ± SEM of *N* = 5. (**F**) Mass photometry of 300 nM ORF1p mixed with 50 nM RNA of different lengths. RNA with ≥90 nt provides sufficient space for two ORF1p trimers to bind. *N* = 3. (**G**) Fluorescence anisotropy of ORF1p binding to 1 nM 6-FAM-labeled 30-nt RNA, 30-nt ssDNA, or 30-bp dsDNA. The binding curve was fitted with a Hill equation, means ± SEM of *N* = 3. (**H**) Binding curves comparing binding of ORF1p to dsDNA [green, same as in (G)] versus ORF1p that was first saturated with 100 nM unlabeled 30-nt RNA and then bound to 1 nM 6-FAM–labeled 30-bp dsDNA (black). Prebound RNA reduced ORF1p’s affinity for dsDNA. Means ± SEM; −RNA: *N* = 3; +RNA: *N* = 4.

L1 encodes two proteins, ORF1p and ORF2p (*10*), that drive a copy-and-paste retrotransposition process (Fig. 1A). While both proteins are required for retrotransposition of L1, the role of ORF1p in the L1 life cycle is poorly understood (*11*, *12*). ORF2p is an endonuclease and reverse transcriptase that catalyzes the insertion of a new copy of L1 into the host genome (*2*, *13*). ORF1p has a low-nanomolar affinity for RNA and has been proposed to be an RNA chaperone (*14*–*16*). Upon translation of the 6-kb L1-mRNA, a dynamic assembly of multiple ORF1p trimers envelops the RNA and a small number of ORF2 proteins in a ribonucleoprotein (RNP) condensate (*12*, *17*, *18*). This RNP formation is essential for transposon activity in vivo (*19*, *20*), but its functional role remains unclear.

ORF1p is composed of an N-terminal intrinsically disordered region (IDR), followed by a long coiled-coil (CC) domain and a globular C-terminal domain (CTD) containing the RNA recognition motif (RRM; Fig. 1A) (*21*–*24*). IDRs often facilitate multivalent interactions by electrostatic, polar and hydrophobic interactions (*25*–*27*); the ORF1p IDR was shown to be required for condensate formation (*19*, *20*). In addition, the charge and position of a basic patch within the IDR are crucial for condensate formation (*19*) and retrotransposition activity in cells (*12*, *19*, *28*, *29*). The IDR contains two highly conserved phosphorylation sites that are also required for retrotransposition activity (*30*, *31*).

The ORF1p CC consists of 14 heptad repeats (*32*) that are interrupted by a three–amino acid stammer at positions 91 to 93 that has been proposed to increase CC flexibility (*28*, *33*). Interaction of the CC domains facilitates formation of a dumbbell-shaped ORF1p homotrimer (*11*, *21*, *23*), which has previously been resolved by crystallography and nuclear magnetic resonance (NMR) spectroscopy in parts (*28*, *34*, *35*). ORF1p also assembles into higher oligomeric states (*36*, *37*) of unclear structure; determining the nature of trimer-trimer interactions is crucial to resolve this question. We previously studied the processes of phase separation and condensation of ORF1p in vitro and in human cells, respectively, and established condensate formation as critical for L1 retrotransposition (*19*). However, the role of ORF1p oligomerization in L1 RNP assembly and retrotransposition remains unclear.

Oligomerization has been shown to affect ORF1p’s interactions with nucleic acids (*36*), which is of great interest given the requirement of L1 proteins to interact with both RNA and DNA during retrotransposition. Beyond its high affinity for RNA (*36*, *38*), ORF1p has also been shown to bind to DNA in both bulk (*36*) and single-molecule experiments (*37*, *39*). This raises the possibility that ORF1p could be involved in DNA target site recognition or priming. Previously, DNA binding activity was investigated with ORF1p alone, but never within assembled RNP complexes. As a result, it is unknown whether this DNA binding capability is restricted to the isolated protein or could be extended to the ORF1p-RNP and whether ORF1p has another role in retrotransposition besides RNA protection.

Here, we combine single-molecule DNA curtain (*40*) technology, mass photometry (*41*), and fluorescence anisotropy with high-resolution cell imaging to reveal the interplay between protein oligomerization and nucleic acid binding, RNP assembly and its localization to DNA, and L1 retrotransposition. We found that ORF1p forms multimers of trimers. Trimer multimerization is driven by electrostatic interactions and tuned by RNA binding. Furthermore, we generated RNP condensates by mixing L1 RNA and ORF1p and determined that these RNP condensates bind to DNA. By altering RNP composition, we show that excess ORF1p is required to generate double-stranded DNA (dsDNA) binding RNP condensates, consistent with the high stoichiometry of ORF1p observed in cells (*12*). Mutational studies suggest a connection between oligomerization behavior and stable DNA binding of RNP condensates. We present that L1 RNP condensates colocalize with mitotic chromatin in cells and can only gain access to the nucleus to successfully complete retrotransposition if they interact with DNA. Together, our study establishes direct DNA binding as an additional property of L1 RNP condensates. Thus, we provide insights into how ORF1p oligomerization and nucleic acid interactions are interdependent in RNP condensate formation and target site recognition and determine a crucial role for ORF1p-RNP condensates to initiate L1 retrotransposition.

## RESULTS

### RNA binding influences ORF1p oligomerization

We investigated the oligomerization states of purified human ORF1p using single-molecule mass photometry (*41*–*43*). First, we analyzed the mass distribution of 300 nM ORF1p in a 50 mM KCl buffer and detected only minor amounts of trimer but a high proportion of hexamers and peaks for higher-order oligomeric forms (Fig. 1B and fig. S1A). We found that with lower protein concentration, the proportion of hexamers decreased while the proportion of trimers increased (Fig. 1C). The same trend was observed with increasing salt concentrations. This suggests that oligomerization is driven by both protein concentration and electrostatic interactions. ORF1p monomers were hardly detected, which may be due to the detection limit of the mass photometry device (about 40 kDa) or because ORF1p is known to primarily assemble into a stable trimer (*35*, *36*).

To examine the effect of RNA binding on ORF1p oligomerization, we added a 30-nt fragment of the 5′ untranslated region (5′UTR) of L1-RNA to ORF1p. With increasing RNA concentrations, ORF1p oligomers dissociated into trimeric species (Fig. 1D and fig. S1A), suggesting that trimer-trimer interactions are disturbed by RNA binding. The RNA-induced dissociation of ORF1p hexamers can be modeled by a Hill equation with a Hill coefficient of 2.1 ± 0.3, consistent with a cooperative binding model where RNA binding on one trimer facilitates the dissociation of the hexamer releasing another RNA binding site on the second trimer (Fig. 1E). RNA binding also induced a mass shift of both the trimer and the hexamer, suggesting that both species are able to bind to RNA (Fig. 1D). In agreement with previous studies (*36*), our results demonstrate that RNA binding influences ORF1p oligomerization.

With a twofold excess of ORF1p (100 nM ORF1p trimer + 50 nM RNA), only the trimer peak was observed in mass photometry, suggesting that the 30-nt RNA provided space for only one ORF1p trimer to bind (Fig. 1F). We increased the RNA length to 60 nt and still observed only one trimer on the RNA. Upon further increasing the RNA length to 90 nt, we began to observe a second peak, likely due to two ORF1p trimers associated with the same RNA molecule (Fig. 1F). This peak became even more prominent at 120-nt RNA length. Thus, while 30-nt RNA are sufficient for one ORF1p trimer to bind (*35*, *36*), an RNA length of 90 nt provides enough space to accommodate two trimers. This suggests that a short 15-nt RNA linker between the ORF1p trimers is required to allow multiple ORF1p to assemble on one RNA molecule (*35*).

To support these findings, we modeled RNA binding of an ORF1p trimer using AlphaFold 3 (fig. S1B) (*44*). The RNA binding site is located at a strongly positively charged cleft between the RRM and the CTD, which can open and close upon substrate binding (*24*, *34*, *35*). In five computed models of the trimer, the RNA was wrapped around the three CTDs and interacted with each binding cleft of the individual monomers as postulated previously (*35*, *45*). We found that the 30-nt RNA is fully occupied by one trimer, which again implies that a short RNA spacer is required for beads-on-a-string-like assembly of ORF1p on RNA.

### RNA and dsDNA bind at the same site on the ORF1p trimer

The exact binding mode of different nucleic acids to the ORF1p trimer is unknown as no nucleic acid–bound structure is available. The curved and narrow binding cleft suggests a preference for flexible and unstructured nucleic acids (*35*). We determined the binding affinities of ORF1p to different nucleic acid substrates using fluorescence anisotropy. To this end, we titrated unlabeled ORF1p to fluorescently labeled 30-nt RNA, 30-nt single-stranded DNA (ssDNA), or 30–base pair (bp) dsDNA. The resulting binding curves (Fig. 1G) showed the highest affinity to RNA [dissociation constant (K_d_) = 3.9 ± 1.1 nM], followed by ssDNA (K_d_ = 8.3 ± 1.8 nM) and dsDNA (K_d_ = 18.9 ± 1.3 nM), in agreement with previous studies (*35*, *36*). In addition, we tested binding of ORF1p to alternative nucleic acid substrates and found no significant difference in affinity between RNA and a double-stranded RNA/DNA hybrid (K_d_ = 4.7 ± 0.6 nM) or a polyadenylate [poly(A)] (K_d_ = 6.0 ± 1.2 nM; fig. S1C). Note that RNA affinity was slightly reduced at higher salt concentrations (fig. S1D).

To examine whether reduced binding affinity of ORF1p toward dsDNA originates from a different binding position than RNA, we repeated the mass photometry experiments in the presence of increasing concentrations of 30-bp dsDNA. We observed that dsDNA induces hexamer dissociation in a manner comparable to RNA (fig. S1E), suggesting that dsDNA binds at the same site on ORF1p as RNA. To further test this hypothesis, we performed a competition assay using fluorescence anisotropy. We saturated ORF1p with 100 nM RNA and monitored subsequent binding to 1 nM labeled dsDNA (Fig. 1H). Once ORF1p is occupied by RNA, the affinity for dsDNA drops by 33-fold, suggesting that dsDNA and RNA compete for the same binding site on ORF1p. Similar results were obtained when first saturating ORF1p with 100 nM dsDNA and then adding 1 nM labeled RNA to the sample (fig. S1F).

We modeled binding of dsDNA to ORF1p trimer using AlphaFold 3 (fig. S1G) and observed a different binding mode compared to RNA. As dsDNA is more rigid, it is unable to wrap around the ORF1p trimer and thus forms different contacts with the protein than RNA. We hence conclude that although dsDNA and RNA bind to the same site on ORF1p, their binding mode is different.

### Excess protein enables ORF1p-RNP condensate formation and binding to DNA

So far, we have investigated ORF1p binding to small oligos to study the details of ORF1p trimer interactions with nucleic acids. However, in a naturally occurring ORF1p-RNP, many ORF1p trimers cotranslationally assemble onto a long L1 mRNA. Therefore, we studied ORF1p binding to a 2-kb fragment of L1 RNA as our subsequent model. Interactions between ORF1p and these longer RNAs led to the formation of RNP condensates, which we previously found to be essential for L1 retrotransposition (*19*). There are multiple possible reasons why enveloping L1 RNA in an ORF1p condensate facilitates the L1 life cycle. We decided to test the hypothesis that these RNP condensates might bind to dsDNA.

**Fig. 2.**
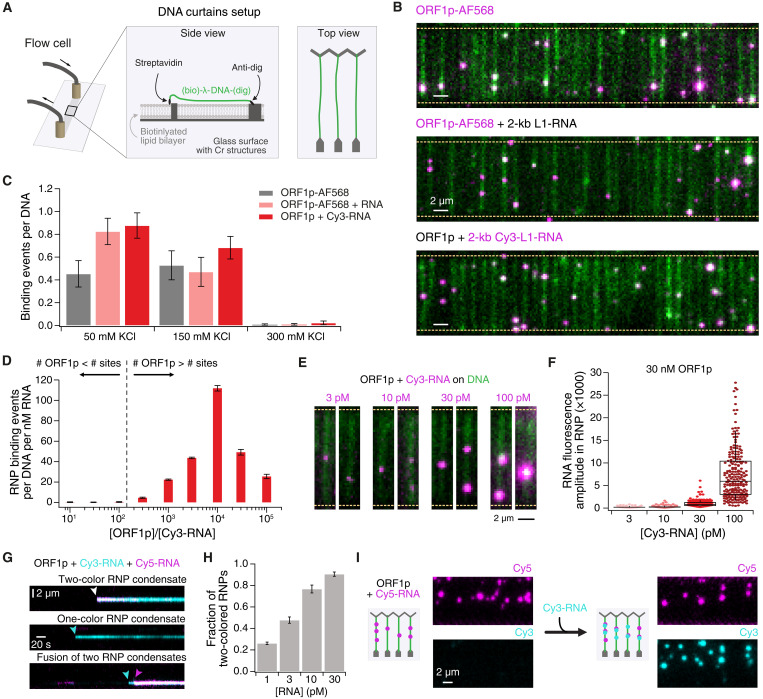
RNP condensates formed by ORF1p and a 2-kb L1 RNA fragment bind efficiently to DNA curtains. (**A**) Schematic representation of the DNA curtains experimental setup. Lipid-bound λ-phage DNA is introduced into a custom-built flow cell that contains chromium structures as diffusion barriers. Flow causes the λ- DNA to stretch across the chromium barrier and anchor to a second chromium structure, thus holding the DNA in a stretched configuration. (**B**) TIRF microscopy of ORF1p or ORF1p-RNP condensates (magenta) binding to YOYO-1-labeled λ-DNA (green). Yellow dashed lines illustrate Cr barrier position. Top: 30 nM ORF1p-AF568; middle: 30 nM ORF1p-AF568 preincubated with 30 pM 2-kb L1-RNA; bottom: 30 nM unlabeled ORF1p preincubated with 30 pM 2-kb Cy3-labeled L1-RNA. (**C**) Average number of binding events per DNA strand at different KCl concentrations. Means ± SEM, *N* ≥ 3. (**D**) Weighted average of RNP condensate binding events per DNA strand per nM RNA at 150 mM KCl. The dashed line marks the 130× excess of ORF1p that is predicted to saturate all ORF1p trimer binding sites on RNA. Means ± SEM, *N* = 3. (**E**) TIRF microscopy images of ORF1p-RNP condensates (magenta) containing different amounts of Cy3-L1-RNA binding to DNA curtains (green). (**F**) Fluorescence amplitudes of DNA-bound RNP condensates at 150 mM KCl. Unlabeled ORF1p (30 nM) was mixed with increasing amounts of Cy3-L1-RNA, *N* ≥ 3. (**G**) Kymograms of DNA binding events of 30 nM ORF1p premixed with 15 pM Cy3-RNA (cyan) and 15 pM Cy5-RNA (magenta). (**H**) Fraction of RNP condensates containing two RNA colors at different total RNA concentrations. Cy3-RNA and Cy5-RNA were added at an equimolar concentration. Means ± SEM, *N* = 4. (**I**) ORF1p was preincubated with Cy5-RNA (magenta). Cy3-RNA (cyan) was then added to the flow cell and was recruited to already bound RNP condensates.

We used single-molecule DNA curtains, which consist of arrays of lambda phage DNA (λ-DNA) stretched between two chromium barriers in a microfluidic flow cell (Fig. 2A) (*40*, *46*). In this system, each individual fluorescently labeled dsDNA molecule can be directly visualized, and binding of fluorescently labeled molecules can be monitored in real time using total internal reflection fluorescence (TIRF) microscopy.

We found that fluorescently labeled ORF1p alone [Fig. 2B (top) and fig. S2A] and ORF1p complexed with a 2kb RNA as an RNP (Fig. 2B, middle) both bind to dsDNA curtains. In the absence of RNA, ORF1p forms large clusters that bind to DNA curtains; these clusters are larger at higher concentrations (fig. S2B). Strikingly, RNPs formed from unlabeled ORF1p and fluorescently labeled RNA also bound to dsDNA curtains (Fig. 2B, bottom). This implies that, while RNA-bound ORF1p trimers have low affinity for dsDNA (Fig. 1H), large numbers of ORF1p trimers condensed with a long RNA do bind dsDNA. We labeled both protein and RNA and quantified the frequency of colocalization between the two signals. ORF1p was part of an RNP condensate in almost all cases (fig. S2C), indicating that ORF1p preferentially assembles into RNP condensates, even when it is in high stoichiometric excess. Furthermore, this interaction is very stable as more than 60% of the bound RNP condensates remained bound to DNA for more than 5 min (fig. S2D).

We next quantified binding of ORF1p alone and ORF1p-RNP condensates at different salt concentrations. At 50 mM KCl, RNP binding was enhanced compared to ORF1p, but at 150 mM KCl, binding was similar (Fig. 2C). Neither species bound at 300 mM KCl, indicating that binding to DNA relies upon electrostatic interactions. Cy3-RNA alone did not bind to DNA curtains (fig. S2A, top right). Therefore, we used RNP condensates containing labeled RNA and unlabeled ORF1p for the following experiments to ensure that the observed binding events were only attributed to RNP condensates.

To investigate the optimal ratio between protein and RNA in an RNP condensate, we determined the number of binding events on DNA while keeping either RNA or ORF1p concentration constant and varying the other component. We performed these measurements at 150 mM KCl to approximate physiological conditions and still collect reasonable amounts of data. For better data interpretation, we normalized the data with the respective RNA concentration as the RNA carries the label (Fig. 2D, unnormalized data; fig. S2, E and F). Our mass photometry data suggests that a 2-kb RNA provides a maximum of 44 direct binding sites for ORF1p trimers (45-nt RNA are required for each trimer). This analysis predicts that a 130-fold molar excess of protein would saturate the RNA, and further ORF1p binding beyond this stoichiometry (super-saturation) would be in a different mode, for example, through protein-protein interactions. We did not observe any RNP condensate binding to DNA with an ORF1p:RNA stoichiometric excess below 130-fold (Fig. 2D). At these stoichiometries, every ORF1p nucleic acid–binding cleft can be occupied by RNA and would therefore be unable to bind to DNA, similar to the scenario in Fig. 1H. In support of this idea, when we mixed ORF1p in 1:1 molar ratio with short RNA oligos, we also did not observe any DNA binding (fig. S2A, bottom right). In contrast, when the ORF1p concentration was in 1000-fold excess, RNP condensate binding to DNA was robust and further increased with higher protein concentrations to a maximum binding efficiency with 10,000-fold excess (Fig. 2D). These results suggest that RNP condensates can be recruited to DNA if a high excess of ORF1p trimers super-saturates the L1 RNA molecule and provides additional ORF1p with unoccupied nucleic acid–binding sites to the condensate.

### ORF1p-RNP condensates are variable in composition

During our measurements, it became apparent that RNPs become brighter with increasing RNA concentrations (Fig. 2, E and F). This indicates that ORF1p-RNP condensates can, in principle, incorporate more than one RNA molecule. To assess this hypothesis, we mixed ORF1p with two differently colored RNAs. Not only did this yield two-colored RNP condensates (Fig. 2G), but the fraction of two-colored RNP condensates also increased to 90.5 ± 0.1% as we increased the RNA concentration to 30 pM at an equal ratio of the two colors (Fig. 2H). Furthermore, RNP condensates that are already bound to DNA can recruit additional RNA molecules (Fig. 2I), suggesting that DNA-bound RNP condensates are still multivalent. Supporting this idea, we also observed fusion of RNP condensates. We increased the incubation time of protein and RNA from 1 min to 10 or 30 min and found a strong increase in fluorescence (fig. S2G), demonstrating that with more time, larger condensates can form. The same was observed when mixing differently colored preformed RNP condensates before loading them onto the DNA. In 56.5 ± 11.3% of the cases, we found two-colored fused RNP condensates (fig. S2H).

### ORF1p-RNP condensates target A/T-rich DNA regions

The lambda-phage genome we used for the DNA curtains assay contains 12 instances of the known L1 target site TTTTTAA (*13*, *47*–*50*) and is extended such that protein-binding positions are colinear with the DNA sequence. To evaluate whether ORF1p-RNPs have a preferred binding site on DNA, we analyzed binding positions and found that they align, to some extent, with the target site and in general with A/T-rich DNA regions (fig. S2I). This suggests that ORF1p-RNPs could play a role in target site recognition and L1 stabilization on genomic DNA.

### The ORF1p N terminus is required for the DNA binding activity of RNPs

**Fig. 3.**
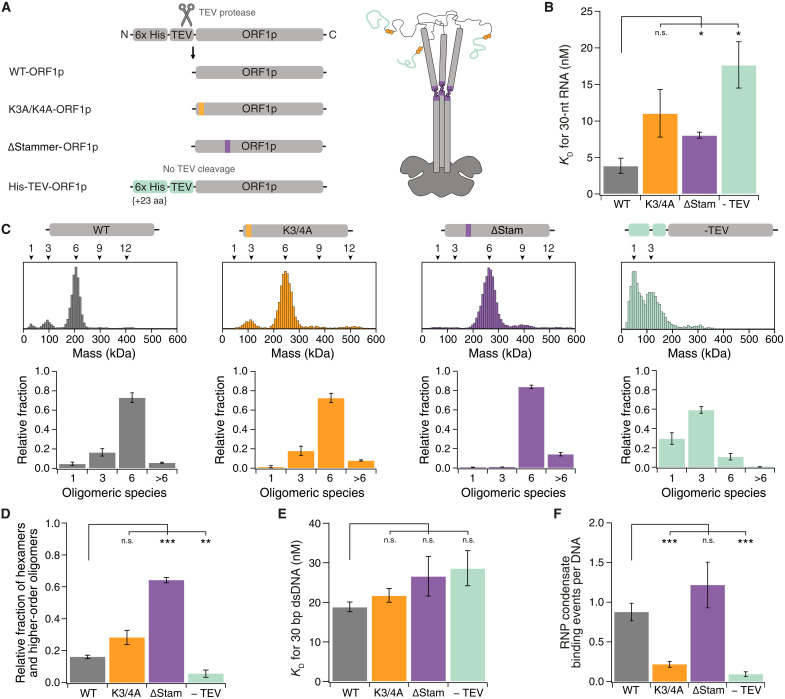
ORF1p mutants exhibit different oligomerization and nucleic acid-binding properties than the wildtype. (**A**) Schematic of ORF1p mutants showing the location of the mutations within the homo-trimer. His-TEV-ORF1p was not digested by TEV protease resulting in a 23–amino acid (aa) elongated N terminus (-TEV). (**B**) Dissociation constants of 30-nt RNA binding of ORF1p variants determined by fluorescent anisotropy. Means ± SEM. Significance determined by Student’s *t* test. WT: *N* = 3; K3A/K4A: *N* = 3, *P* = 0.15; ΔStammer: *N* = 3, *P* = 0.041; -TEV: *N* = 4, *P* = 0.018. (**C**) Mass photometry of ORF1p variants. Top: Histogram of molecular masses. Bottom: Relative fraction of oligomeric species (1: monomer, 3: trimer, 6: hexamer, >6: higher-order oligomers). Means ± SEM. WT: *N* = 7; K3A/K4A: *N* = 6; ΔStammer: *N* = 4; -TEV: *N* = 5. (**D**) Relative fraction of hexamers and higher-order oligomers of ORF1p mutants (300 nM) at 150 mM KCl determined by mass photometry. Means ± SEM. Significance determined by Student’s *t* test. WT: *n* = 3; K3A/K4A: *N* = 5, *P* = 0.054; ΔStammer: *N* = 3, *P* = 6.6·10^−6^; -TEV: *N* = 5, *P* = 0.0077. (**E**) Dissociation constants for ORF1p variants binding to 1 nM 30-bp dsDNA were determined by fluorescence anisotropy. Means ± SEM of *N* = 3. Significance determined by Student’s *t* test. K3A/K4A: *P* = 0.252; ΔStammer: *P* = 0.259; -TEV: *P* = 0.151. (**F**) Average number of binding events of 30 nM ORF1p mutants mixed with 30pM Cy3-L1-RNA on DNA curtains at 50 mM KCl. Means ± SEM. Significance determined by Student’s *t* test. WT: *N* = 14; K3A/K4A: *N* = 7, *P* = 3.9 × 10^−5^; ΔStammer: *N* = 4, *P* = 0.33; -TEV: *N* = 4, *P* = 6.4 × 10^−6^. n.s., not significant.

As RNP formation is influenced by both ORF1p oligomerization and nucleic acid interaction, we wondered whether we could assign these activities to distinct regions within the ORF1p structure. To this end, we created ORF1p mutants (Fig. 3A) with either a mutation of the N-terminal basic patch (K3A/K4A-ORF1p), a deletion of the CC stammer motif (ΔStammer-ORF1p), or an elongated N terminus (His-TEV-ORF1p).

We determined the RNA binding affinity of the three mutants and found only a slight increase in K_d_ compared to wild type (WT) (Fig. 3B). Next, we investigated their oligomerization state using mass photometry and found no change in oligomerization of K3A/K4A-ORF1p but a slight stabilization of the hexamer and higher-order oligomers for the ΔStammer-ORF1p (Fig. 3C). This stabilization is even more pronounced at 150 mM KCl (Fig. 3D), consistent with the concept that the CC sequence affects intratrimer configurations that influence oligomerization (*37*). The N-terminally elongated mutant (His-TEV-ORF1p) displayed strongly impaired oligomerization (Fig. 3, C and D). It appeared that even trimer formation was affected, as monomers were observed in 30% of all counts. This is likely a lower-bound estimate of the fraction of monomers, as the mass of an ORF1p monomer (40 kDa) is close to the lower detection limit of the mass photometer (*51*). With an N-terminal elongation, contact points between N-terminal amino acids and the CC domain that are essential for trimer-trimer interactions (*20*) are probably lost, leading to impaired oligomerization.

All ORF1p mutants had comparable DNA binding affinities as determined by fluorescence anisotropy (Fig. 3E). However, His-TEV-ORF1p RNP condensates showed almost no binding to DNA curtains (Fig. 3F). Thus, the N-terminal elongation specifically abrogates binding of the RNP condensate to DNA, perhaps because the organization of the condensate is altered due to the impaired oligomerization behavior of this mutant. ΔStammer-ORF1p RNP condensates showed similar binding compared to WT, but K3A/K4A-ORF1p-RNP condensates bound significantly worse than WT on DNA curtains (Fig. 3F), although hexamer formation and RNA binding were not affected by this mutation. Basic patches, especially in IDRs, form electrostatic interactions with the phosphate backbone of RNA (*52*), and long RNA molecules can themselves modulate the chemical environment of this disordered regions to favor RNP complex assembly (*53*–*55*). As K3A/K4A-RNP condensates are less DNA binding competent, we speculate that the basic patches of multiple IDRs form a second binding site for nucleic acids, which then promote further RNP condensate assembly. This would also explain the reduced DNA binding of His-TEV-ORF1p-RNP condensates as, in this variant, the position of the basic patch is shifted (*12*, *19*, *28*, *29*), possibly preventing the formation of this additional nucleic acid binding site. Together, these results show that the N terminus of ORF1p is critical for the DNA binding of ORF1p-RNP condensates.

### The DNA binding activity of ORF1p is necessary for L1 chromosomal association and retrotransposition

**Fig. 4.**
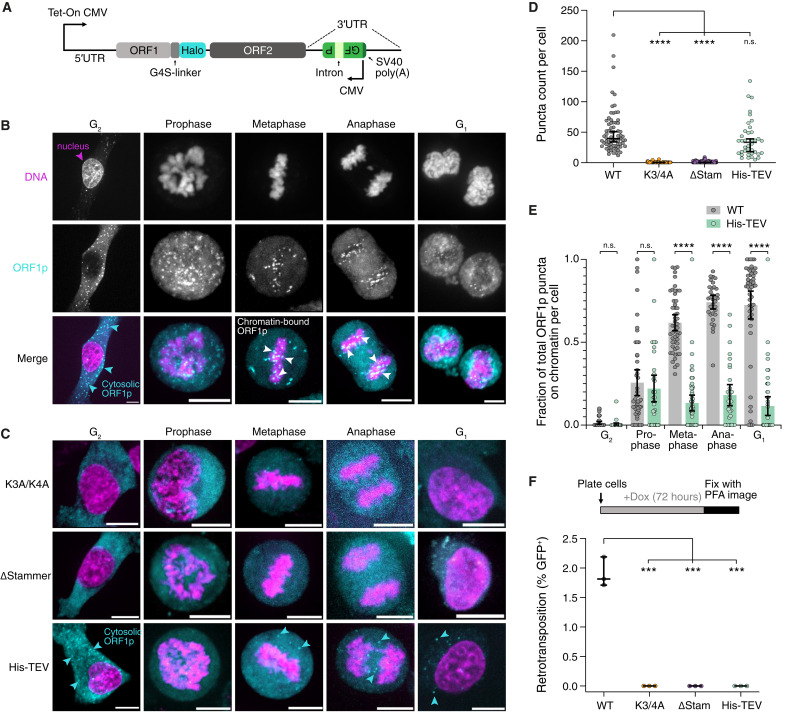
Binding of ORF1p puncta to mitotic chromatin is required for L1 chromosome association and retrotransposition. (**A**) Schematic of Tet-inducible L1 expression construct with a retrotransposition reporter. ORF1p carries a HaloTag for visualization. GFP-AI reporter requires transcription, removal of the intron by splicing, and subsequent retrotransposition for GFP expression. (**B**) WT-ORF1p puncta bind mitotic chromatin following nuclear envelope breakdown and associate with chromosomes throughout mitosis. HeLa cells expressing WT-ORF1p-Halo were treated with doxycycline for 12 hours, treated with SiR-DNA to visualize DNA and JFX549 to label ORF1p, and then imaged. The DNA signal (top), WT-ORF1p-Halo signal (middle), and merged image (bottom) are shown for various cell cycle stages (left to right). Representative maximum intensity projections of Z-stacks are shown. (**C**) K3A/K4A-ORF1p and ΔStammer-ORF1p were unable to form puncta, while His-TEV-ORF1p formed puncta that did not bind mitotic chromatin. Representative images of each mutant (top to bottom) for various cell cycle stages (left to right) are shown. The same imaging paradigm was performed as in (B). (**D**) WT- and His-TEV-ORF1p robustly form puncta, but K3A/K4A-ORF1p and ΔStammer-ORF1p did not. Significance determined by Student’s *t* test. *n* > 30 per cell cycle stage; *N* = 3. (**E**) Quantification of the colocalization between ORF1p puncta and chromatin at various stages of the cell cycle for WT-ORF1p and His-TEV-ORF1p. The number of puncta colocalized with chromatin was divided by the total number of puncta in each cell. Significance determined by multiple Student’s *t* tests. *n* > 30 per cell cycle stage, *N* = 3. (**F**) Top: Cells were continuously treated with doxycycline for 72 hours, and retrotransposition rates were calculated using the GFP-AI reporter. Bottom: The percentage of cells expressing GFP (GFP^+^) obtained by imaging was scored for each ORF1p mutant in addition to WT. Significance determined by Student’s *t* test. *n* > 400, *N* = 3.

Given the ability of ORF1p to bind DNA both on its own and within the context of an RNP condensate, we wondered if we could observe binding of ORF1p to DNA in cells and if this activity is relevant to L1 retrotransposition activity. We used a previously described Tet-inducible, active, endogenous-like L1 expression construct in which ORF1p was fused at its C terminus to a HaloTag (ORF1p-Halo) (Fig. 4A) (*56*). This enabled visualization of ORF1p in live cells by labeling with Janelia Fluor HaloTag Ligand JFX549 (*57*). The construct also contained a reporter to assess the ability of this engineered construct and its variants to complete the full L1 retrotransposition cycle. The reporter consisted of a green fluorescent protein (GFP) cassette interrupted by an antisense γ-globin intron (GFP-AI) in the 3′UTR of our construct (*11*, *58*, *59*). The antisense disrupts the GFP open reading frame such that transcription, splicing, and successful retrotransposition of this spliced L1 mRNA are required for subsequent expression of an uninterrupted GFP coding sequence from a novel insertion site. This reporter enabled us to relate changes in ORF1p behavior upon mutation to the completion of the L1 retrotransposition life cycle.

Consistent with our prior results, the formation of ORF1p puncta was observed after around 6 hours of induction of our expression system with doxycycline. We compared WT-ORF1p puncta formation to that of the mutants we had already assessed in vitro: K3A/K4A-ORF1p, ΔStammer-ORF1p, and His-TEV-ORF1p (Fig. 4, B to D). At early time points, WT-ORF1p and His-TEV-ORF1p formed a similar number of puncta. However, neither the K3A/K4A-ORF1p nor the ΔStammer-ORF1p mutants formed puncta (Fig. 4D). This result is unexpected, especially for ΔStammer-ORF1p, as this mutant showed stable oligomerization, nucleic acid binding, and efficient RNP condensate formation in vitro (Fig. 3, B and C). We hypothesize that the stammer motif may be important for a specific cotranslational condensate assembly pathway that enables efficient L1 RNP condensate formation in the presence of high concentrations of competing cytoplasmic RNA in cells.

The WT-ORF1p puncta were mostly localized to the cytoplasm but sometimes also to the nucleus (Fig. 4B). To determine whether the ORF1p puncta contain RNA, we performed HCR RNA-FISH (hybridized chain reaction RNA fluorescence in situ hybridization) (*60*) and found that cytosolic WT-ORF1p puncta often colocalized with L1 RNA (fig. S3A and B). This indicates that WT-ORF1p forms RNP condensates with L1 RNA in the cytosol. While there were minimal nuclear puncta in G_2_ before nuclear envelope breakdown, we observed substantial colocalization of ORF1p with chromatin upon nuclear envelope breakdown in prometaphase. This colocalization increased during metaphase and anaphase and persisted into the subsequent G_1_ (Fig. 4, B and E, and fig. S3, C, E, and F). This is consistent with prior reports that ORF1p binds to chromosomes during mitosis as a result of nuclear envelope breakdown and is then retained in the daughter nuclei upon nuclear envelope reassembly post-division (*11*). We additionally noted correlation between the motion of the ORF1p puncta and mitotic chromatin, suggesting binding to the DNA as a mechanism for L1 nuclear entry.

Expression of the N-terminally elongated His-TEV-ORF1p mutant produced puncta that were colocalized with L1 RNA, suggesting that this mutant can also form RNP condensates (fig. S3, A and B). However, the His-TEV ORF1p puncta had reduced colocalization with mitotic chromatin and diminished nuclear puncta in G_1_ relative to WT (Fig. 4, C and E, and fig. S3, D, E, and G). To assess the role of L1 RNP’s DNA binding activity in L1 retrotransposition, we induced WT and mutant ORF1p-expressing HeLa cells with doxycycline for 72 hours and looked for GFP expression, indicative of successful retrotransposition events (Fig. 4A). While about 2% of the cells that expressed WT-ORF1p underwent retrotransposition, similar to our prior observations (*19*), no retrotransposition events were observed in cells expressing His-TEV-ORF1p or the other two mutants (Fig. 4F). These data support the hypothesis that the DNA binding activity of super-saturated ORF1p condensates is necessary for L1 retrotransposition. Together, the results from our cell experiments suggest a model where L1 RNP condensates bind DNA following nuclear envelope breakdown during mitosis to enable the L1 RNP condensate to access the genome and carry out retrotransposition.

## DISCUSSION

### DNA binding of ORF1p-RNP condensates enables binding to mitotic chromosomes

Here, we described DNA binding of L1 RNP condensates composed of RNA and ORF1p. Our use of RNP condensate generation and single-molecule imaging advance our understanding and are complementary to previous studies of isolated ORF1p (*36*, *37*, *39*). Our data indicate that while isolated ORF1p has the ability to bind DNA, its comparatively higher RNA affinity causes it to preferentially assemble on RNA into RNP condensates (Fig. 2). As ORF1p binds cotranslationally to L1 mRNA in cells (cis preference) (*22*, *61*), we propose that this initial RNP formation could serve multiple functions in the cytoplasm including melting of RNA secondary structure as shown for other high-affinity RNA binders (*62*, *63*) and protection of the L1 mRNA from the innate immune system (*64*–*66*). After translation, the RNP condensate must gain access to the nucleus to allow retrotransposition on genomic target sites. As the RNP complex is likely several hundred nanometers in diameter, it is too large to enter the nucleus through the nuclear pore (*67*, *68*). As also reported for several retroviruses (*69*), our study is consistent with a model where L1 gains access to the genome by binding chromosomes when the nuclear envelope is disassembled during mitosis (*11*). The ability of L1 RNP condensates to directly bind DNA in vitro (Fig. 2) and the observation that these particles also bind mitotic chromatin (Fig. 4) suggest that the L1 RNP condensate acts as a transport vehicle to bring L1 RNA into the nucleus and into proximity to known integration sequences on DNA.

### L1 RNP condensates evade mechanisms that exclude large cytoplasmic particles from the nucleus during mitosis

During nuclear envelope breakdown it is crucial that cytoplasmic and nuclear contents stay separated. Sufficiently small particles can be exported through nuclear pores in G_1_, but large particles cannot be restored to their correct compartment by this mechanism. Therefore, mitotic chromosomes are coated with proteins, including Ki-67 and barrier-to-autointegration factor (BAF) (*70*) that prevent large cytoplasmic macromolecular objects from entering into the chromatin (*71*). Preventing invasive molecules such as retrotransposons from gaining access to the genome is an essential host-defense mechanism. Given that our results suggest that L1 RNP condensates bind to mitotic chromatin, we speculate that L1 RNP condensates can overcome this Ki-67– and BAF-mediated nuclear exclusion. Perhaps, the condensates have biophysical properties that allow them to partition into the perichromosomal layer formed by Ki-67 during mitosis (*70*, *72*, *73*) or that enable association with dense chromatin networks that are formed by BAF during nuclear envelope reformation (*71*, *74*).

### ORF1p may guide RNP condensates to L1 target sites

Our experiments on generated RNP condensates suggest that ORF1p could play a role in L1 target site acquisition. Recently, a study structurally resolved ORF2p binding to the DNA target site showing a very tightly regulated recognition of the target sequence TTTTTAA (*50*). For ORF1p, no sequence-specific nucleic acid binding has been reported (*38*). We observed only a slight target site preference, but ORF1p-RNP condensates did get enriched on A/T-rich regions of DNA (fig. S2I). Therefore, we hypothesize that ORF1p guides the RNP condensate toward A/T-rich regions, within which ORF2p then has a higher probability to specifically recognize the L1 integration target sequence.

Besides DNA binding, ORF1p is known for its nucleic acid chaperone activity, inducing DNA melting, strand annealing, and strand exchange (*14*, *36*, *39*, *75*, *76*). These processes are more likely to occur at A/T-rich regions where base pair interactions are weaker (*77*). In accordance, we observed most ORF1p and ORF1p-RNP condensate binding events on DNA at A/T-rich regions, and these binding events were very static (fig. S2A), which would be consistent with the abovementioned models that postulate local DNA melting by ORF1p. Supporting this idea, fluorescence anisotropy data showed that ORF1p has a high affinity for dsRNA/DNA hybrids (fig. S1C), comparable to the affinity for single-stranded nucleic acids, suggesting that ORF1p might melt these hybrids. In addition, the high affinity for RNA/DNA hybrids points toward a possible involvement of ORF1p in hybridization of L1 RNA with the genomic target site and other strand annealing processes during the reverse transcription mechanism (*48*, *75*).

### ORF1p stoichiometry modulates L1 RNP condensate function

**Fig. 5.**
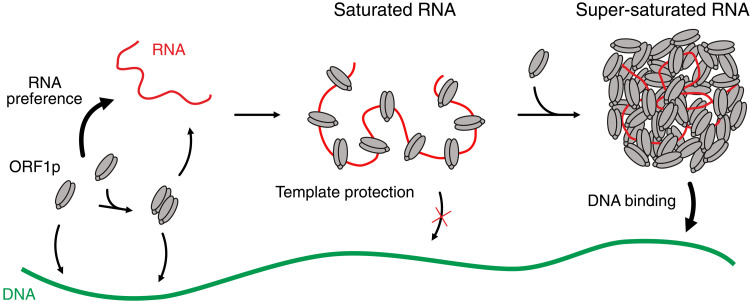
Model for ORF1p RNP condensate formation and DNA recognition. ORF1p forms homo-trimers and higher-order oligomers that can bind to DNA but have a clear preference for RNA (left). Upon RNA binding, ORF1p trimers occupy first all available binding sites on the RNA, which protects the RNA from degradation (middle) but is unable to bind to DNA. Continued ORF1p accumulation leads to super-saturation of the RNA, which is required to form a DNA binding–competent RNP condensate (right).

Strikingly, ORF1p RNP condensates have no DNA binding activity until ORF1p has super-saturated the RNA. As ORF1p has a high affinity and preference for RNA, it initially binds the RNA tightly by wrapping it around the trimer occupying all three binding clefts on the ORF1p trimer, which does not allow for additional dsDNA binding (Fig. 1H). However, with high ORF1p excess, the RNP condensate properties change such that additional dsDNA binding is possible (Fig. 2C). We think that, upon super-saturation, additional ORF1p trimers are recruited through protein-protein interactions, which are not in contact with the L1 RNA, and hence provide free nucleic acid binding sites (Fig. 5). As mutations in the N-terminal IDR affected RNP condensate formation in vitro (Fig. 3F) and in vivo (Fig. 4C), and the IDRs are known to promote condensation (*19*, *20*), we propose that this domain enables the formation of a super-saturated, DNA binding competent RNP condensate.

A very recent study also showed that ORF1p excess is required for supplemental nucleic acid binding (*78*). The authors suggest that upon super-saturation, additional ORF1p recruitment leads to a partial dissociation of prebound ORF1p from the RNA chain, liberating one binding cleft of the trimer, which can then bind additional nucleic acid molecules. In this model, super-saturation weakens the trimer-RNA interaction (facilitated dissociation) so that one trimer can bind multiple nucleic acids at the same time (*78*). Our data in fig. S1F imply that dsDNA saturation of ORF1p may still allow some RNA binding (small shoulder in the binding curve), and the AlphaFold 3 model predicts that dsDNA only occupies one binding cleft in an ORF1p trimer (fig. S1G). Furthermore, ORF1p’s chaperone activity would require the simultaneous binding of multiple nucleic acids on one ORF1p trimer (*14*, *75*). Thus, we hypothesize that both additional protein-protein interaction mode and facilitated dissociation may synergize to enable DNA binding by super-saturated L1 RNP condensates.

In our study, the ΔStammer mutant formed no puncta in cells although RNA binding, oligomerization, and RNP condensate formation in vitro were unaffected. We speculate that changes in the CC domain might lead to less tightly packed RNP condensates that can be attacked by ribonucleases or the innate immune system of the host cell, e.g., by association with stress granules (*29*, *79*). Another possibility is that cotranslational assembly of the RNP condensates might be affected by this mutation as it was shown before that the stammer is essential for ORF1p recruitment to L1 RNPs (*79*).

We speculate that the emergence of DNA binding activity only upon formation of super-saturated RNPs is helpful to ensure on-target activity of ORF1p. In this model, the high affinity of ORF1p for RNA might cause it to be sequestered by the general cellular RNA pool, but these substoichiometric amounts of ORF1p on incorrect RNA targets would not be able to target DNA and would hence be relatively inert. Only ORF1p cotranslationally assembled at high stoichiometry on the L1 mRNA would gain DNA binding activity. Recent evidence suggests that ORF1p-RNP condensates indeed assemble predominantly in cis on the mRNA from which they were translated (*19*).

Together, our results suggest that ORF1p could confer distinct functions to the L1 RNP condensate depending on stoichiometry. This provides a kinetic control of L1 RNP condensate function during cotranslational assembly. At the beginning of translation, excess RNA disrupts the formation of ORF1p trimer-trimer contacts and favors a configuration, where the L1 RNA is sequestered in the RNA binding cleft, perhaps providing protection against degradation or avoiding detection by the innate immune system. With continued translation, ORF1p will super-saturate the L1 RNA, and excess nucleic acid binding sites render RNP condensates competent to bind to mitotic chromatin at potential integration sites. Thus, L1 condensates have the remarkable feature that an additional property—DNA binding—emerges at a threshold stoichiometry of ORF1p. It will be interesting to see if other RNP condensates undergo functional changes when their component stoichiometries change.

## MATERIALS AND METHODS

### Experimental design

This study aims to gain mechanistic insights into ORF1p-mediated L1-RNP condensate formation by combining single-molecule mass photometry and DNA curtains experiments with cellular assays.

### ORF1p bacterial expression constructs

The His-TEV-ORF1p bacterial expression construct was purchased in pET28a from Genewiz, Azenta Life Science containing an N-terminal His-tag and TEV cleavage site and a C-terminal ybbR-tag. All other constructs were made previously (*19*).

#### pLH2087: WT-ORF1p (Homo sapiens)

GMGKKQNRKTGNSKTQSASPPPKERSSSPATEQSWMENDFDELREEGFRRSNYSELREDIQTKGKEVENFEKNLEECITRITNTEKCLKELMELKTKARELREECRSLRSRCDQLEERVSAMEDEMNEMKREGKFREKRIKRNEQSLQEIWDYVKRPNLRLIGVPESDVENGTKLENTLQDIIQENFPNLARQANVQIQEIQRTPQRYSSRRATPRHIIVRFTKVEMKEKMLRAAREKGRVTLKGKPIRLTADLSAETLQARREWGPIFNILKEKNFQPRISYPAKLSFISEGEIKYFIDKQMLRDFVTTRPALKELLKEALNMERNNRYQPLQNHAKM.

#### pLH2075: K3AK4A-ORF1p

GMG**AA**QNRKTGNSKTQSASPPPKERSSSPATEQSWMENDFDELREEGFRRSNYSELREDIQTKGKEVENFEKNLEECITRITNTEKCLKELMELKTKARELREECRSLRSRCDQLEERVSAMEDEMNEMKREGKFREKRIKRNEQSLQEIWDYVKRPNLRLIGVPESDVENGTKLENTLQDIIQENFPNLARQANVQIQEIQRTPQRYSSRRATPRHIIVRFTKVEMKEKMLRAAREKGRVTLKGKPIRLTADLSAETLQARREWGPIFNILKEKNFQPRISYPAKLSFISEGEIKYFIDKQMLRDFVTTRPALKELLKEALNMERNNRYQPLQNHAKM.

Bold: Mutation site.

#### pLH2036: ΔStammer-ORF1p

GMGKKQNRKTGNSKTQSASPPPKERSSSPATEQSWMENDFDELREEGFRRSNYSELREDIQTKGKEVENFEKNLEECITRITNTEKCLKE**LK**TKARELREECRSLRSRCDQLEERVSAMEDEMNEMKREGKFREKRIKRNEQSLQEIWDYVKRPNLRLIGVPESDVENGTKLENTLQDIIQENFPNLARQANVQIQEIQRTPQRYSSRRATPRHIIVRFTKVEMKEKMLRAAREKGRVTLKGKPIRLTADLSAETLQARREWGPIFNILKEKNFQPRISYPAKLSFISEGEIKYFIDKQMLRDFVTTRPALKELLKEALNMERNNRYQPLQNHAKM.

Bold: Between these residues, the three stammer amino acids M91, E92, and L93 were deleted.

#### pET28a: His-TEV-ORF1p

MKHHHHHHPMSDYDIPTT**ENLYFQ****G**MGKKQNRKTGNSKTQSASPPPKERSSSPATEQSWMENDFDELREEGFRRSNYSELREDIQTKGKEVENFEKNLEECITRITNTEKCLKELMELKTKARELREECRSLRSRCDQLEERVSAMEDEMNEMKREGKFREKRIKRNEQSLQEIWDYVKRPNLRLIGVPESDVENGTKLENTLQDIIQENFPNLARQANVQIQEIQRTPQRYSSRRATPRHIIVRFTKVEMKEKMLRAAREKGRVTLKGKPIRLTADLSAETLQARREWGPIFNILKEKNFQPRISYPAKLSFISEGEIKYFIDKQMLRDFVTTRPALKELLKEALNMERNNRYQPLQNHAKMGSDSLEFIASKLA.

Underlined sequence: In all other constructs, this sequence is cut off during TEV protease digestion; bold: TEV cleavage sequence. The construct contains a C-terminal ybbR-tag.

### ORF1p mammalian expression constructs

The His-TEV-ORF1p mammalian expression construct was made using Gibson assembly of a Not I– and Psh AI–digested pLH2035 backbone with two overlapping polymerase chain reaction products containing the His-TEV sequence and homology to the backbone, as well as a single-stranded homology template obtained from IDT. All other constructs used were made previously (*19*). All sequences were verified using long-read nanopore sequencing (Plasmidsaurus) or Sanger sequencing (Genewiz).

#### pLH2035: WT-ORF1p (H. sapiens)

MGKKQNRKTGNSKTQSASPPPKERSSSPATEQSWMENDFDELREEGFRRSNYSELREDIQTKGKEVENFEKNLEECITRITNTEKCLKELMELKTKARELREECRSLRSRCDQLEERVSAMEDEMNEMKREGKFREKRIKRNEQSLQEIWDYVKRPNLRLIGVPESDVENGTKLENTLQDIIQENFPNLARQANVQIQEIQRTPQRYSSRRATPRHIIVRFTKVEMKEKMLRAAREKGRVTLKGKPIRLTADLSAETLQARREWGPIFNILKEKNFQPRISYPAKLSFISEGEIKYFIDKQMLRDFVTTRPALKELLKEALNMERNNRYQPLQNHAKMGGGGSAEIGTGFPFDPHYVEVLGERMHYVDVGPRDGTPVLFLHGNPTSSYVWRNIIPHVAPTHRCIAPDLIGMGKSDKPDLGYFFDDHVRFMDAFIEALGLEEVVLVIHDWGSALGFHWAKRNPERVKGIAFMEFIRPIPTWDEWPEFARETFQAFRTTDVGRKLIIDQNVFIEGTLPMGVVRPLTEVEMDHYREPFLNPVDREPLWRFPNELPIAGEPANIVALVEEYMDWLHQSPVPKLLFWGTPGVLIPPAEAARLAKSLPNCKAVDIGPGLNLLQEDNPDLIGSEIARWLSTLEISG.

Underlined sequence: G4S-linker + HaloTag.

#### pLH2042: K3A/K4A-ORF1p

MG**AA**QNRKTGNSKTQSASPPPKERSSSPATEQSWMENDFDELREEGFRRSNYSELREDIQTKGKEVENFEKNLEECITRITNTEKCLKELMELKTKARELREECRSLRSRCDQLEERVSAMEDEMNEMKREGKFREKRIKRNEQSLQEIWDYVKRPNLRLIGVPESDVENGTKLENTLQDIIQENFPNLARQANVQIQEIQRTPQRYSSRRATPRHIIVRFTKVEMKEKMLRAAREKGRVTLKGKPIRLTADLSAETLQARREWGPIFNILKEKNFQPRISYPAKLSFISEGEIKYFIDKQMLRDFVTTRPALKELLKEALNMERNNRYQPLQNHAKMGGGGSAEIGTGFPFDPHYVEVLGERMHYVDVGPRDGTPVLFLHGNPTSSYVWRNIIPHVAPTHRCIAPDLIGMGKSDKPDLGYFFDDHVRFMDAFIEALGLEEVVLVIHDWGSALGFHWAKRNPERVKGIAFMEFIRPIPTWDEWPEFARETFQAFRTTDVGRKLIIDQNVFIEGTLPMGVVRPLTEVEMDHYREPFLNPVDREPLWRFPNELPIAGEPANIVALVEEYMDWLHQSPVPKLLFWGTPGVLIPPAEAARLAKSLPNCKAVDIGPGLNLLQEDNPDLIGSEIARWLSTLEISG.

Bold: Mutation position; underlined sequence: G4S-linker + HaloTag.

#### pLH2040: ΔStammer-ORF1p

MGKKQNRKTGNSKTQSASPPPKERSSSPATEQSWMENDFDELREEGFRRSNYSELREDIQTKGKEVENFEKNLEECITRITNTEKCLKE**LK**TKARELREECRSLRSRCDQLEERVSAMEDEMNEMKREGKFREKRIKRNEQSLQEIWDYVKRPNLRLIGVPESDVENGTKLENTLQDIIQENFPNLARQANVQIQEIQRTPQRYSSRRATPRHIIVRFTKVEMKEKMLRAAREKGRVTLKGKPIRLTADLSAETLQARREWGPIFNILKEKNFQPRISYPAKLSFISEGEIKYFIDKQMLRDFVTTRPALKELLKEALNMERNNRYQPLQNHAKMGGGGSAEIGTGFPFDPHYVEVLGERMHYVDVGPRDGTPVLFLHGNPTSSYVWRNIIPHVAPTHRCIAPDLIGMGKSDKPDLGYFFDDHVRFMDAFIEALGLEEVVLVIHDWGSALGFHWAKRNPERVKGIAFMEFIRPIPTWDEWPEFARETFQAFRTTDVGRKLIIDQNVFIEGTLPMGVVRPLTEVEMDHYREPFLNPVDREPLWRFPNELPIAGEPANIVALVEEYMDWLHQSPVPKLLFWGTPGVLIPPAEAARLAKSLPNCKAVDIGPGLNLLQEDNPDLIGSEIARWLSTLEISG.

Bold: Between these residues, the 3 stammer residues M91, E92, L93 were deleted; underlined sequence: G4S-linker + HaloTag.

#### pLH2722: His-TEV-ORF1p

**HHHHHHPMSDYDIPTTENLYFQG**MGKKQNRKTGNSKTQSASPPPKERSSSPATEQSWMENDFDELREEGFRRSNYSELREDIQTKGKEVENFEKNLEECITRITNTEKCLKELMELKTKARELREECRSLRSRCDQLEERVSAMEDEMNEMKREGKFREKRIKRNEQSLQEIWDYVKRPNLRLIGVPESDVENGTKLENTLQDIIQENFPNLARQANVQIQEIQRTPQRYSSRRATPRHIIVRFTKVEMKEKMLRAAREKGRVTLKGKPIRLTADLSAETLQARREWGPIFNILKEKNFQPRISYPAKLSFISEGEIKYFIDKQMLRDFVTTRPALKELLKEALNMERNNRYQPLQNHAKMGGGGSAEIGTGFPFDPHYVEVLGERMHYVDVGPRDGTPVLFLHGNPTSSYVWRNIIPHVAPTHRCIAPDLIGMGKSDKPDLGYFFDDHVRFMDAFIEALGLEEVVLVIHDWGSALGFHWAKRNPERVKGIAFMEFIRPIPTWDEWPEFARETFQAFRTTDVGRKLIIDQNVFIEGTLPMGVVRPLTEVEMDHYREPFLNPVDREPLWRFPNELPIAGEPANIVALVEEYMDWLHQSPVPKLLFWGTPGVLIPPAEAARLAKSLPNCKAVDIGPGLNLLQEDNPDLIGSEIARWLSTLEISG.

Bold: Additional residues to elongate the N terminus; underlined sequence: G4S-linker + HaloTag.

### ORF1p expression and purification

WT-ORF1p and K3AK4A-ORF1p were expressed in BL21(DE3) cells and purified as described previously (*19*). Protein labeling was done using Alexa Fluor 568 *N*-hydroxysuccinimide–ester dye followed by size exclusion chromatography as described (*19*).

His-TEV-ORF1p and ΔStammer-ORF1p constructs were transformed into *Escherichia coli* LOBSTR expression strain (Kerafast). Cultures were grown in LB medium, induced with 100 μM isopropyl-β-d-thiogalactopyranoside and cultivated for 15 hours at 16°C. After harvesting, cells were resuspended in denaturating lysis buffer [20 mM Hepes (pH 8.0), 500 mM NaCl, 6 M urea, 20 mM imidazole, 5% glycerol, and 1 mM dithiothreitol (DTT)] and lysed by sonication. Lysate was centrifuged for 30 min at 20,000 rpm, and the supernatant was loaded on Ni–nitrilotriacetic acid beads (Macherey-Nagel). Beads were washed with 2 M urea in wash buffer [20 mM Hepes (pH 8.0), 500 mM NaCl, 20 mM imidazole, 5% glycerol, and 1 mM DTT] followed by further washing with stepwise reduction of urea concentration (0.5 and 0 M urea). The protein was eluted with 5 ml of elution buffer [20 mM Hepes (pH 8.0), 500 mM NaCl, 200 mM imidazole, 5% glycerol, and 1 mM DTT] and concentrated to 500 μl using Amicon Ultra-15 filters [30 kDa molecular weight cut-off (MWCO), Merck]. For TEV cleavage of ΔStammer-ORF1p, the protein sample was mixed with 4 U of TEV protease (New England Biolabs) per nanomole of protein and incubated overnight at 4°C while rotating. The proteins were further purified by size exclusion chromatography using a Superose 6 Increase 10/300 GL (Cytiva) in gel filtration buffer [20 mM Hepes (pH 7.5), 500 mM KCl, 5% glycerol, and 1 mM DTT] and stored afterward at −70°C.

### ORF1p AlphaFold model

Structure prediction of WT-ORF1p bound to 30-nt RNA or 30-bp dsDNA (sequences see table S1) was performed using AlphaFold 3 (*44*). Illustration was done using ChimeraX (*80*).

### Nucleic acid constructs

#### Labeled 2-kb RNA for DNA curtains assay

The 1970-nt-long RNA construct was produced via in vitro transcription of the ORF1 gene using Cy3- or Cy5-labeled uridine 5′-triphosphates as described previously (*19*).

#### Short-nucleic acids for mass photometry and fluorescence anisotropy

Thirty-nucleotide-long nucleic acid constructs were purchased from Metabion or Biomers. Sequences can be found in table S1.

A 30-nt RNA was selected from 5′UTR of human LINE-1, which does not contain secondary structures. The 30-nt ssDNA construct contains the same sequence such as the RNA, and the 30-bp dsDNA construct was produced by hybridizing the 30-nt ssDNA construct with a reverse complement sequence (30-nt rv oligo). The success of hybridization was verified by tris-borate EDTA–polyacrylamide gel electrophoresis. For fluorescence anisotropy measurements, all constructs were purchased with a 5′-6-FAM fluorescent label. For 6-FAM-dsDNA construct, 6-FAM-30–nt ssDNA was hybridized with an unlabeled reverse complement sequence.

The RNA/DNA hybrid was produced by hybridizing 6-FAM–labeled 30-nt RNA with the reverse complement DNA oligo (30-nt rv oligo) used also for dsDNA production.

RNA long constructs (60 to 120 nt) were produced by in vitro transcription. A DNA template with the respective length was produced from an empty pRS424 vector including a T7 promoter. RNA was then produced using the HiScribe T7 High Yield RNA Synthesis Kit (NEB) for 5 hours at 37°C followed by deoxyribonuclease I treatment for 15 min at 37°C and an RNA purification using the Monarch RNA Cleanup Kit (NEB). Sequences can be found in table S1.

### Mass photometry

Mass photometry of protein samples was performed using the OneMP and the TwoMP mass photometer (Refeyn). For measurements, microwell gaskets were placed on a glass coverslip to form a well for 20 μl of sample. Mass photometry buffer [20 mM Hepes (pH 7.5), 50 mM KCl, 1 mM MgCl_2_, and 1 mM DTT, filtered with 0.2 μm] was used for all experiments unless otherwise stated. After focusing, the protein sample was added to the buffer droplet. Contrasts of individual protein binding events on the glass surface were recorded for 1 min using Acquire software (Refeyn). For protein–nucleic acid interactions, protein and nucleic acid were mixed in 20 μl of mass photometry buffer and incubated for 5 min at room temperature. The focusing of the device occurred, in this case, buffer-free before adding the premixed sample and starting the measurement. Standard proteins of known mass [bovine serum albumin (BSA) (Sigma-Aldrich), ovalbumin, and aldolase (gel filtration HMW calibration kit, Cytiva)] were used for mass calibration.

With Discover software (Refeyn), measured contrasts were transformed into protein masses using the calibration curve. The collected masses were plotted in a histogram with 100 bins and a bin width of 8 kDa to visualize the mass distribution using Igor Pro 8 software. To determine the appearance of different oligomeric states, histogram peaks were fitted by Gaussian distributions, and the area under the curve was determined. Relative appearance was then calculated by dividing individual areas by the sum of all areas. The mean appearance of oligomeric states was determined from at least three independent experiments. The average mass of each oligomeric states was determined from the peak position of the Gaussian distribution of at least three independent experiments. The values can be found in tables S2 to S4. To visualize low-abundant higher-order oligomeric states of ORF1p, histograms of mass distributions were additionally filtered with a quadratic polynomial and a span of nine using the Savitzky-Golay method.

### Fluorescence anisotropy

For fluorescence anisotropy measurements, 1 nM 6-FAM–labeled oligo was mixed with increasing concentrations of ORF1p in a final volume of 30 μl in a black 384-well plate (Greiner Bio-One). Measurement buffer [20 mM Hepes (pH 7.5), 50 mM KCl, 1 mM MgCl_2_, and 1 mM DTT] was used unless otherwise stated. After mixing of protein and oligo, the plate was centrifuged for 1 min at 700 rpm, and fluorescence polarization and resulting anisotropy were measured using the Tecan infinite M-1000 plate reader (Tecan).

For data analysis, the value of the protein-free control was subtracted from all other values, which were then normalized setting the smallest value to zero and the biggest value to one using custom-written software in Igor Pro 8 software (Wavemetrics). Fitting a hill equation resulted in a K_d_ value. Mean K_d_ was calculated from at least three independent experiments.

### DNA curtains setup

For DNA curtains assay, custom-made flow cells with Cr diffusion barriers were produced as described previously (*40*). The flow cell was connected to a microfluidic system by nanoports glued on drilled holes in the fused silica slide (UQG Optics). TIRF microscopy was performed using a prism-type Nikon Ti2 microscope equipped with an electron multiplying charge-coupled camera (Andor iXon life). Samples were excited with 488-, 561-, and 640-nm lasers (OBIS LX/LS, coherent), and videos were recorded in NIS elements (Nikon). The beam splitter HC BS 640 (AHF Analysetechnik) with additional filters for green (585/65 BP, Thorlabs) and red channel (700/75 BP, Thorlabs) was used in an OptoSplit II (Cairn Research).

Flow cells were prepared as described elsewhere (*46*). In brief, a lipid bilayer (dioleoylphosphatidylcholine, DOPE, and DOPE-biotin, Otto Nordwald) was formed on the glass surface, followed by the addition of digoxigenin-binding protein DIG10.3, which attaches randomly at the chromium pedestals. Next, unspecific binding sites were blocked by BSA, and streptavidin was added to the biotinylated lipids. Last, biotinylated DNA was added to attach to the lipids.

As a DNA substrate, lambda phage DNA (NEB) was used. For attachment to the lipid bilayer, oligos containing biotin (5′-P-aggtcgccgccc-bio-3′) or digoxigenin (5′-P-gggcggcgacct-dig-3′) were hybridized to the cos sites of the lambda DNA, ligated overnight at room temperature using T4 ligase (NEB), and purified using HiPrep 16/60 Sephacryl S-300 HR column (Cytiva) in TE150 buffer.

### DNA curtains experiments

All experiments were performed in running buffer [20 mM Hepes (pH 7.5), BSA (1 mg/ml), 1 mM MgCl_2_, and 1 mM DTT] containing 0.5 nM YOYO-1 dye for DNA staining and 50, 150, or 300 mM KCl. Samples were diluted in running buffer without YOYO-1. An oxygen scavenging system was used in sample and running buffer containing glucose oxidase (Carl Roth), catalase (Sigma-Aldrich), and 0.8% glucose. All protein-containing samples were added to the flow cell via an injection valve and incubated for 5 min on the DNA curtains before flushing out all unbound proteins. DNA was imaged with a 488-nm laser at 5 mW and an illumination rate of 2.5 s.

Protein and RNP samples were imaged with either a 561-nm laser (Alexa Fluor 568– or Cy3-labeled samples) or with a 640-nm laser (Cy5-labeled RNA) at 20 mW and an illumination rate of 500 ms. All results were analyzed using custom written software in Igor Pro 8 (Wavemetrics).

#### Protein and RNP binding with one label

For WT-ORF1p, binding was studied by either adding 30 nM Alexa Fluor 568–labeled WT-ORF1p (ORF1p-AF568) directly to the DNA curtains or by first forming an RNP by mixing 30 nM ORF1p-AF568 with 30 pM 2-kb L1-RNA and incubating for 1 min at room temperature, which was then added to the DNA curtains. Alternatively, 30 nM unlabeled WT-ORF1p was premixed with 30 pM 2-kb Cy3-labeled L1-RNA (Cy3-L1-RNA). Binding was determined at 50, 150, and 300 mM KCl. The number of binding events was counted on two barriers with a field of view for each of 13.5 μm by 138 μm, and this number was divided by the number of DNA strands. An average binding event number per DNA was determined from at least three independent experiments. For the ORF1p variants, 30 nM unlabeled ORF1p were mixed with 30 pM Cy3-L1-RNA, incubated for 1 min, and loaded on the DNA. ORF1p variant binding was determined at 50 mM KCl.

To determine the amount of ORF1p that form RNPs in the presence of RNA, 30 nM ORF1p-AF568 was mixed with 30 pM Cy5-L1-RNA, incubated for 1 min at room temperature and loaded on DNA curtains. The number of two-colored binding events (ORF1p colocalized with L1-RNA) was compared to all observed binding events.

For testing different ratios of ORF1p to RNA, either ORF1p concentration was kept constant at 30 nM and different Cy3-L1-RNA concentrations were added (fig. S2E) or Cy3-L1-RNA concentration was kept constant at 30 pM and different ORF1p concentrations were added (fig. S2F). In both cases, the number of binding events per DNA was divided by the RNA concentration in nanomolar to compare binding events. A weighted average of binding events per DNA per nanomolar RNA was determined from three independent experiments. These experiments were performed at 150 mM KCl. RNA fluorescence amplitudes of RNP condensates were determined by tracking individual binding events and calculating the average fluorescence amplitude per event using custom-written software.

Kymograms were calculated from individual exemplary DNA strands over time. Binding position distributions were determined by counting individual binding events of 30 nM ORF1p-AF568 or 30 nM ORF1p + 30 pM Cy3-L1-RNA at 150 mM KCl and plotting the binding position on the DNA in a histogram with 24 bins and a bin size of 2 kbp.

Survival plot was created from binding event lifetimes using the Kaplan-Meier method (*81*). Error bars were determined by bootstrapping.

#### Two-color RNA experiments

ORF1p (30 nM) was mixed with 15 pM Cy3-L1-RNA and 15 pM Cy5-L1-RNA before adding it the flow cell. Alternatively, 30 nM ORF1p were mixed with 15 pM Cy5-L1-RNA, added to the flow cell, and incubated for 5 min. After flushing out all unbound molecules, 15 pM Cy3-L1-RNA was added to the flow cell, and two-colored RNPs were recorded. These experiments were performed at 50 mM KCl.

#### RNP condensate fusion

Two samples were prepared, one containing 30 nM unlabeled ORF1p and 30 pM Cy3-L1-RNA and one containing 30 nM ORF1p and 30 pM Cy5-L1-RNA. The RNPs condensates were incubated for 5 min at 50 mM KCl, mixed afterward, and directly loaded on DNA curtains. RNPs containing Cy3 fluorescence, Cy5 fluorescence, or both colors (fused) were counted, and the relative fraction was calculated from the sum of all events.

### Mammalian cell culture, live-cell imaging, and retrotransposition assay

All quasi-stable cell lines were generated and maintained as described previously (*19*). For Cdk-1 inhibition, quasi-stable HeLa cells expressing pLH2035 were seeded onto non-coated six-well glass-bottom plates (Cellvis). Twenty-four to 48 hours later, cells were treated with doxycycline hyclate (1 μg/ml; Sigma-Aldrich) and RO-3306 (3.5 μg/ml; Cdk1 inhibitor, MedChem Express), which were added to the conditioned media for about 12 hours to induce L1 expression and arrest the cells in G_2_. The treated cells were then stained with 100 nM HaloTag Ligand JF549 (Promega) or JFX549 (Janelia Labs) and 500 nM Sir-DNA (Cytoskeleton) in conditioned media for 30 to 60 min. The cells were then washed twice with warmed Dulbecco’s phosphate buffered saline (DPBS) (Thermo Fisher Scientific), and warmed fresh complete puromycin media (*19*) was added to the cells. Live-cell imaging was then started immediately to capture cells entering mitosis, or cells were allowed to recover for 10 to 30 min in the fresh media and subsequently fixed with 4% formalin (Thermo Fisher Scientific) for later imaging or immunofluorescence assays.

Live-cell imaging was performed as described previously (*19*) on an Andor Yokogawa CSU-X confocal spinning disc on a Nikon TI Eclipse microscope where the stage temperature and CO_2_ levels in the recirculated air were maintained at 37°C and 5% CO_2_, respectively. Fixed cells were imaged as described previously (*19*) on an Andor Yokogawa CSU-X confocal spinning disc on a Nikon TI Eclipse microscope at room temperature.

Retrotransposition assays were conducted as previously described using quasi-stable HeLa M2 cells transfected with a construct expressing the desired L1RP element with a GFP-AI reporter (*19*). Cells were treated with doxycycline hyclate (1 μg/ml; Sigma-Aldrich) for 72 hours and then stained with 100 nM HaloTag Ligand JFX549 (Janelia Labs) and 500 nM Sir-DNA (Cytoskeleton) for 30 to 60 min. Cells were subsequently fixed with 4% formalin for imaging. Retrotransposition events were visualized using microscopy with subsequent image analysis in Fiji to determine the number of cells expressing GFP (GFP^+^).

### Hybridized chain reaction RNA–fluorescence in situ hybridization

For HCR RNA-FISH, the same probes were used as described previously (*19*), the probes were designed and produced by Molecular Instruments. RNA-FISH was performed according to a previously published protocol (*60*). In brief, cells were seeded and induced with doxycycline for 12 hours, followed by subsequent staining with Halo Ligand JFX549. The cells were then washed with PBS, followed by fixation in freshly diluted 4% paraformaldehyde for 10 min. The cells were washed three times with PBS and permeabilized with 1% Triton X-100 (Thermo Fisher Scientific) in PBS for 10 min at room temperature. The prehybridization of the samples was conducted for 30 min at 37°C using prewarmed hybridization buffer (Molecular Instruments). Probes were diluted to 1 nM in prewarmed hybridization buffer and added to the samples. Samples were incubated in the probe solution for 1 hour in a humidified chamber at 37°C and then washed 4 times for 10 mins each in pre-warmed wash buffer (Molecular Instruments). Samples were washed twice for 5 min in 5× SSCT (5× sodium saline citrate with 0.1% Tween 20) and then incubated in 200 μl of amplification buffer (Molecular Instruments) for 30 min at room temperature. HCR amplifiers (Molecular Instruments) corresponding to each primary probe were heated to 95°C for 90 s, were diluted in amplification buffer, and were added to the samples. The samples were incubated for 1 hour at room temperature in a humidified chamber, washed five times for 10 min each in 5× SSCT, washed once with PBS containing 1 μM Hoechst 33342 (Thermo Fisher Scientific), and then immediately imaged. Cells were imaged as described previously (*19*) on an Andor Yokogawa CSU-X confocal spinning disc on a Nikon TI Eclipse microscope at room temperature.

Analysis of ORF1p-RNA colocalization was performed in three dimensions using Python as previously described (*19*). RNA positive spot positions on the three-dimensional (3D) images were detected with the Laplacian of Gaussian method using the python package scikit-image (skimage.feature.blob_log). Quality checking was performed manually by assessing images and corresponding spots in FIJI. Spot positions were assigned to cellular regions of interest (ROIs) that were generated using Cellpose (*82*) that were also manually vetted for accuracy. Spots outside of the ROIs were excluded from analysis. The average intensity signal of the spot detected initially in the RNA channel (640) was measured in the ORF1p-Halo channel (JFX549). To randomize spots, a count of spots was obtained per cellular ROI and an equivalent number of random positions were selected from the pixel coordinates of each ROI. Since the ROI was drawn in two dimensions, but the spot localizations were in three dimensions, the *z*-channel position for each randomly chosen 2D position within an ROI was selected uniformly from the distribution of *z*-channel positions for the detected spots. Only cytoplasmic spots were selected for analysis as cotranslational assembly occurs in the cytoplasm. Nuclear spots would be expected to reflect nascent RNA transcripts. Spots were determined to be cytoplasmic or nuclear based on the average intensity of the nuclear signal for each spot. Spots classified as nuclear were excluded from subsequent analysis. The intensity of a given spot within each cell was normalized by dividing the intensity in that channel by the median channel intensity of the random spots localized to that same cell ROI. The distributions of the normalized intensities of JFX549 were compared to those of the random spots using a nonparametric Mann-Whitney test in Prism 10 (GraphPad). The code is available on Zenodo (*83*).

#### Statistics and reproducibility

Statistical analysis was performed using Igor Pro 8 (Wavemetrics) or Prism 10 (GraphPad) as described in the figure legends for each experiment. Data were presented as the means ± SEM, and *P* values were determined by unpaired two-tailed *t* test or two-way analysis of variance (ANOVA) where appropriate unless otherwise stated in the figure caption or methods section. Significance was set at *P* < 0.05. The results were reproducible and conducted with established internal controls. Experiments were repeated three or more times and yielded similar results. All samples that met proper experimental conditions were included in the analysis.
