# Supplementary material for: LINE-1 ribonucleoprotein condensates bind DNA to enable nuclear entry during mitosis
Source: Sci Adv. 2025 May 2;11(18):eadt9318. doi: 10.1126/sciadv.adt9318 (PMC12047440; doi:10.1126/sciadv.adt9318)
Supplement: Supplementary file 1 — Figs. S1 to S3 Tables S1 to S4 Legend for data S1 [file sciadv.adt9318_sm.pdf]

Science Advances
AAAS

Supplementary Materials for

LINE-1 ribonucleoprotein condensates bind DNA to enable nuclear entry during mitosis

Sarah Zernia *et al.*

Corresponding author: Liam J. Holt, liam.holt@nyulangone.org; Johannes Stigler, stigler@genzentrum.lmu.de

Sci. Adv. **11**, eadt9318 (2025)
DOI: 10.1126/sciadv.adt9318

The PDF file includes:

Figs. S1 to S3
Tables S1 to S4
Legend for data S1

Other Supplementary Material for this manuscript includes the following:

Data S1

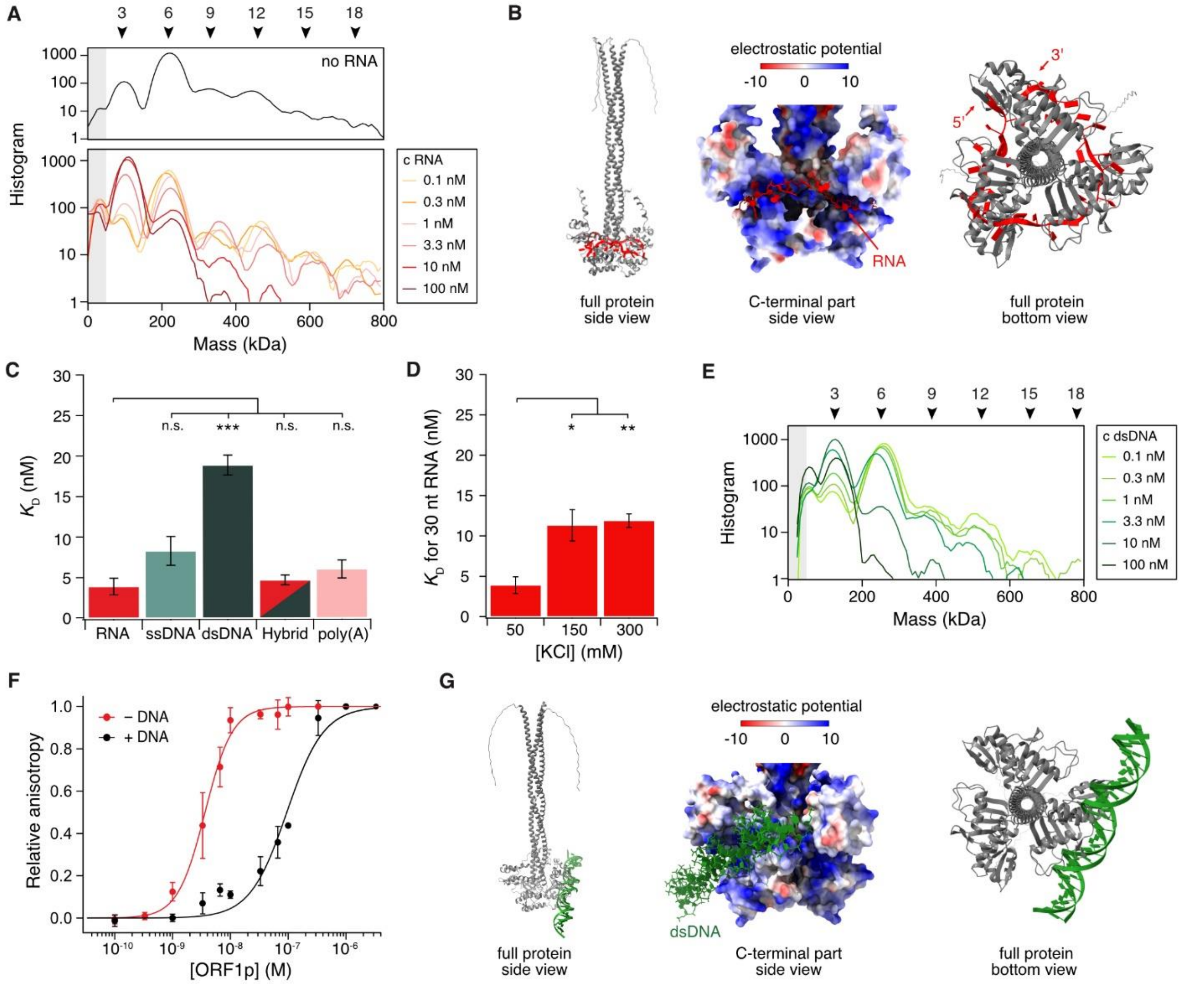

Fig. S1. ORF1p binding to various nucleic acids. (A) Mass photometry histogram of 300 nM ORF1p (top) and 300 nM ORF1p binding to increasing concentrations of RNA oligonucleotide (bottom). To reduce statistical noise, the histogram was smoothed using a Savitzky-Golay filter. Peaks for trimer and higher-order oligomers up to 18-mer could be assigned. **(B)** AlphaFold 3 prediction of ORF1p trimer (grey) binding to 30 nt RNA (red). Left: Full-length ORF1p. Middle: Zoom-in to the C-terminal domain showing the electrostatic surface potential of ORF1p. The RNA is bound in the highly positively charged cleft between CTD and RRM. Right: Bottom-up view of the full-length protein. The RNA is wrapped around the C-terminal part of the ORF1p trimer. **(C)** Dissociation constants of ORF1p binding to different nucleic acid constructs determined by fluorescence anisotropy. Mean ±SEM. Significance was determined by Student's t-test. RNA: N=3; ssDNA: N=3, p=0.12; dsDNA: N=3, p=0.00084; DNA/RNA-hybrid: N=3, p=0.55; poly(A): N=3, p=0.24. **(D)** Dissociation constants for 30 nt RNA binding of ORF1p at different salt concentrations determined by fluorescence anisotropy. Mean ±SEM. Significance was determined by Student's t-test. 50 mM KCl: N=3; 150 mM KCl: N=3, p=0.043; 300 mM KCl: N=3, p=0.0043. **(E)** Mass photometry histogram of 300 nM ORF1p binding to dsDNA, smoothed as in (A). **(F)** Binding curves comparing binding of ORF1p to RNA (red, same as in Fig. 1G) versus ORF1p that was first saturated with 100 nM unlabeled dsDNA and then bound to 1 nM 6-FAM-labeled 30 nt RNA (black). Pre-bound dsDNA reduced ORF1p's affinity for RNA. Mean ±SEM, N=3. **(G)**

AlphaFold 3 prediction of ORF1p trimer (grey) binding to 30 bp dsDNA (green). Left: Full-length ORF1p. Middle: Zoom-in to the C-terminal domain showing the electrostatic surface potential of ORF1p. DNA is only interacting with one ORF1p monomer and also in a different angle than RNA. Right: Bottom-up view of the full-length protein.

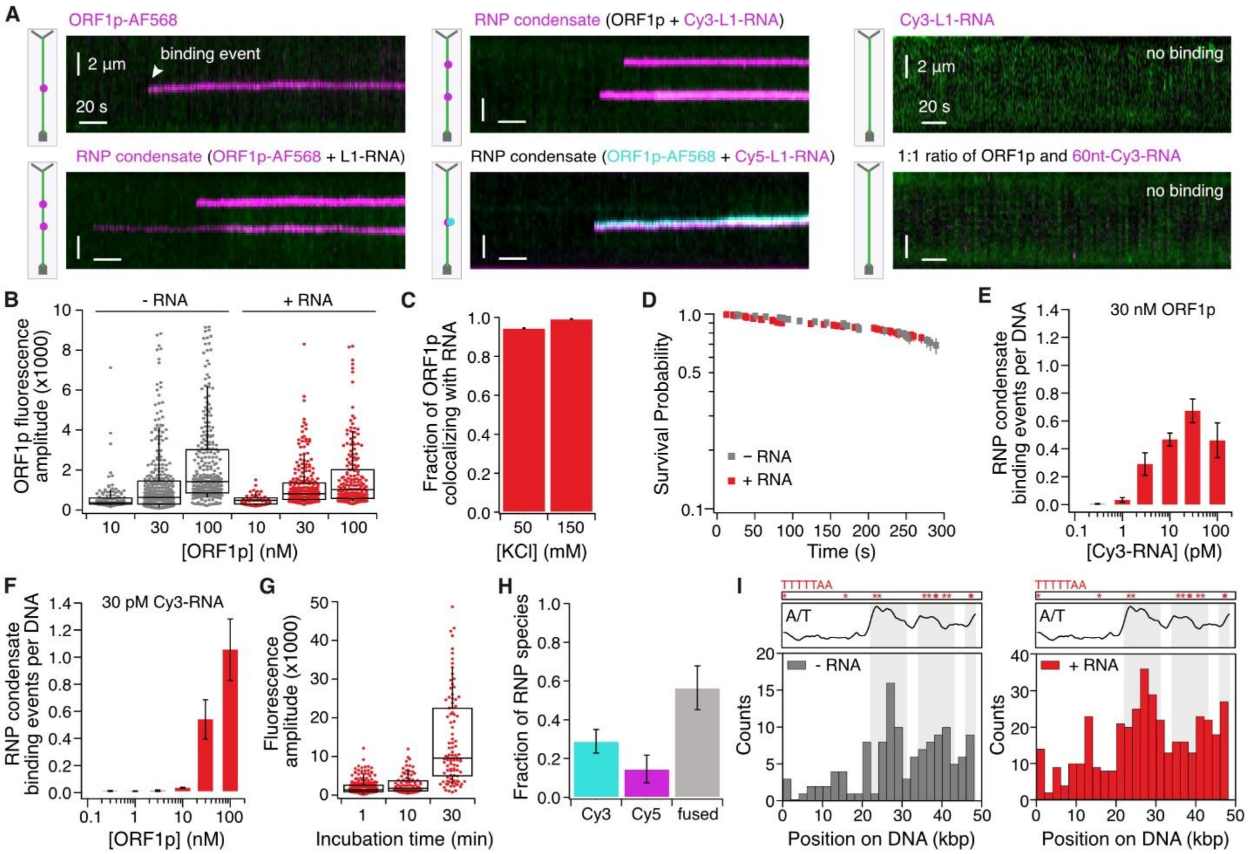

Fig. S2. Binding characteristics of ORF1p-RNP condensates on DNA curtains. (A) Representative kymograms of ORF1p (top left) or RNP condensates (bottom left, middle) binding to DNA. Kymograms showing no binding of 30 pM of 2 kb Cy3-L1-RNA (top right) and 30 nM of 60 nt Cy3-labeled RNA pre-mixed with 30 nM ORF1p (bottom right). **(B)** Fluorescence amplitudes of ORF1p-AF568 binding events on DNA in absence or presence of 2 kb L1-RNA at 150 mM KCl. N≥3. **(C)** Binding events of colocalized 30 nM ORF1p-AF568 and 30 pM Cy5-L1-RNA were counted in comparison to all ORF1p-AF568 binding events on DNA. Mean ± SEM of N=3. **(D)** Survival Plot of ORF1p on DNA in absence or presence of 2 kb L1-RNA at 150 mM KCl. Error bars were determined by bootstrapping. **(E)** Quantification of RNP condensate binding events at constant ORF1p concentration (30 nM) and increasing Cy3-L1-RNA concentration. Mean ± SEM of N=3. **(F)** Quantification of RNP condensate binding events at constant Cy3-L1-RNA concentration (30 pM) and increasing ORF1p concentration. Mean ± SEM of N=3. **(G)** Fluorescence amplitudes of DNA-bound RNP condensates (30 nM ORF1p + 30 pM Cy3-L1-RNA) that were incubated for different lengths of time prior to loading onto DNA curtains. N=3. **(H)** RNPs were formed from ORF1p and L1-RNA molecules labeled with either Cy3 or Cy5. Afterwards, the two samples were mixed and loaded on DNA curtains. The fraction of RNPs that contain only Cy3, only Cy5 or both colors is shown. Mean ± SEM of N=3. **(I)** Binding position of ORF1p on DNA in absence or presence of 2 kb L1-RNA at 150 mM KCl. The position of the L1 target sites and the A/T-content of the λ-DNA is plotted on top of the histograms. -RNA: N=4; +RNA: N=9.

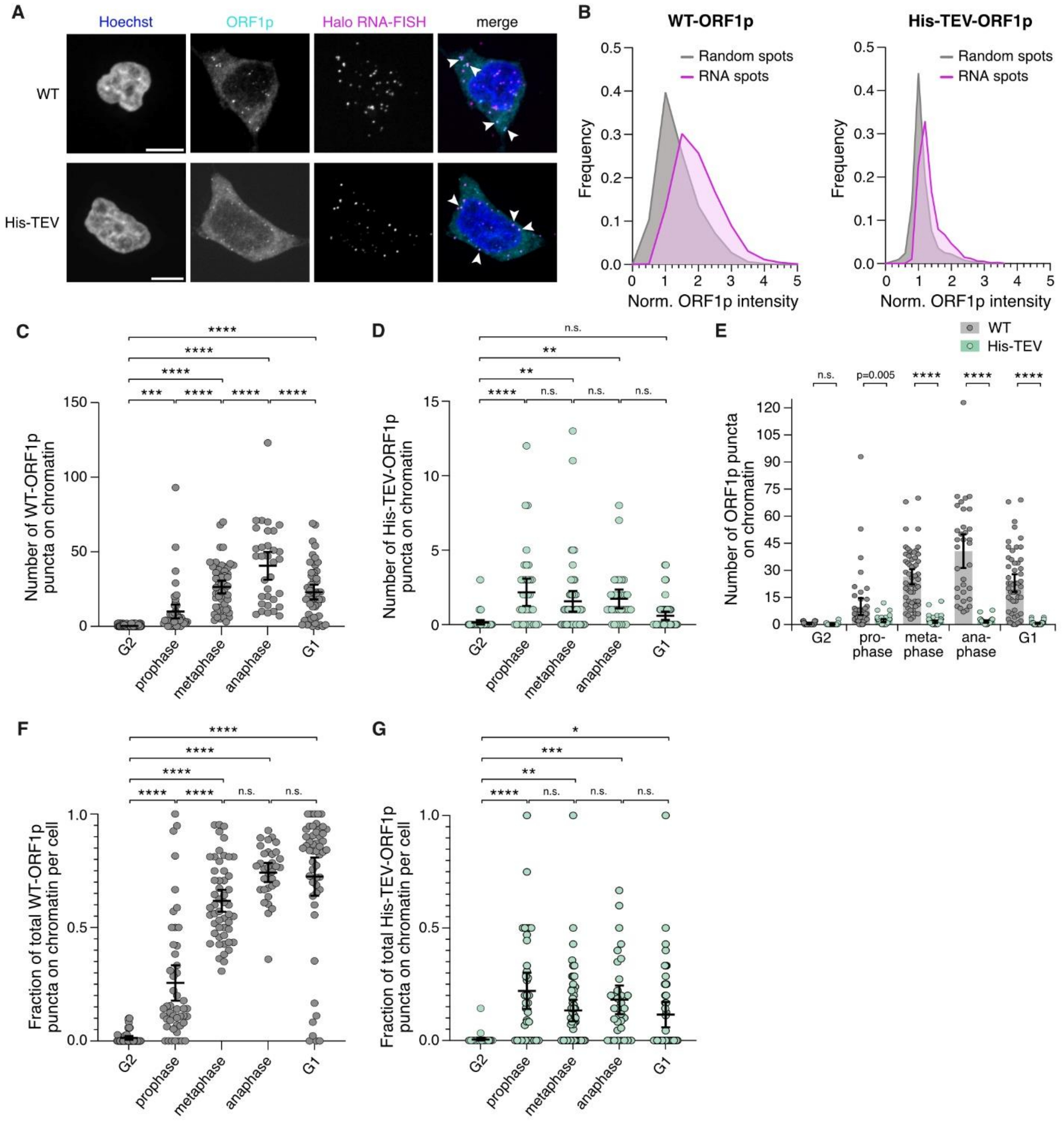

Fig. S3. Puncta dynamics for WT-ORF1p and His-TEV-ORF1p. (A) WT-ORF1p puncta and His-TEV-ORF1p puncta colocalize with L1 RNA. Representative image of a fixed HeLa cell expressing the L1 reporter construct with fluorescently labeled ORF1p-Halo (cyan) and HCR RNA-FISH targeting the HaloTag sequence in the reporter mRNA (magenta). Nuclear staining (Hoechst) is shown in blue in the merged image, and white arrowheads point to colocalized ORF1p-Halo and L1 RNA. Scale bar = 10µm. **(B)** Histograms showing normalized WT-ORF1p (left) or His-TEV-ORF1p (right) fluorescence intensity at detected L1 RNA foci (magenta) compared to an equal number of random cytoplasmic spots per cellular ROI (grey). Significance determined by Mann-Whitney test. WT: two-tailed p-value $p<0.0001$; n = 4529 across 173 cellular

ROIs. His-TEV: two-tailed p-value $p<0.0001$; n = 2180 across 89 cellular ROIs. **(C)** Absolute number of WT-ORF1p puncta colocalized with chromatin increases from prometaphase to anaphase. Significance determined by one-way ANOVA. n>30 per cell cycle stage, N=3. **(D)** Absolute number of His-TEV-ORF1p puncta colocalized with chromatin slightly increases upon nuclear envelope breakdown but remains stable thereafter. Significance determined by one-way ANOVA. n>30 per cell cycle stage, N=3. **(E)** Comparison of absolute numbers of WT- and His-TEV-ORF1p puncta colocalized with chromatin. WT has a significantly greater number of puncta colocalized with chromatin than His-TEV. Significance determined by multiple Student's t-tests. n>30 per cell cycle stage, N=3. **(F)** Fraction of WT-ORF1p puncta colocalized with chromatin per cell across the cell cycle. Same like Fig. 4E, analyzed for significance between cell cycle phases. Significance determined by one-way ANOVA. n>30 per cell cycle stage, N=3. **(G)** Fraction of His-TEV-ORF1p puncta colocalized with chromatin per cell across the cell cycle. Same like Fig. 4E, analyzed for significance between cell cycle phases. Significance determined by one-way ANOVA. n>30 per cell cycle stage, N=3.

Table S1. Nucleic acid sequences of small oligos. 30 bp dsDNA is produced by hybridizing 30 nt ssDNA with 30 nt rv oligo, 30 bp RNA/DNA hybrid is produced by hybridizing 30 nt RNA with 30 nt rv oligo.

**Nucleic acid**	**Sequence (5'-3')**
30 nt RNA	accaucaucaaagaccaaaaguagauaaaa
30 nt ssDNA	ACCATCATCAAAGACCAAAAGTAGATAAAA
30 nt rv oligo	TTTTATCTACTTTTGGTCTTTGATGATGGT
30 nt poly(A)	aaaaaaaaaaaaaaaaaaaaaaaaaaaaaa
60 nt RNA	gggcgaauuggguaccgggcccccccucgaggccgguagaggugugguc aauaa gagcga
70 nt RNA	gggcgaauuggguaccgggcccccccucgaggccgguagaggugugguc aauaaga gcgaccucauacua
80 nt RNA	gggcgaauuggguaccgggcccccccucgaggccgguagaggugugguc aauaaga gcgaccucauacuauaccugagaa
90 nt RNA	gggcgaauuggguaccgggcccccccucgaggccgguagaggugugguc aauaaga gcgaccucauacuauaccugagaaagcaaccuga
100 nt RNA	gggcgaauuggguaccgggcccccccucgaggccgguagaggugugguc aauaaga gcgaccucauacuauaccugagaaagcaaccugaccuacaggaa
110 nt RNA	gggcgaauuggguaccgggcccccccucgaggccgguagaggugugguc aauaaga gcgaccucauacuauaccugagaaagcaaccugaccuacaggaaagaguuacuc
120 nt RNA	gggcgaauuggguaccgggcccccccucgaggccgguagaggugugguc aauaa gagcgaccucauacuauaccugagaaagcaaccugaccuacaggaaagaguuac ucaagaauaaga

Table S2. Average peak position in histograms of mass photometry measurements of ORF1p-WT in absence of presence of RNA. ORF1p: N=4; ORF1p + 3.3 nM RNA: N=5; ORF1p + 100 nM RNA: N=6.

	**ORF1p** Average peak position (± SEM)	**ORF1p + 3.3 nM RNA** Average peak position (± SEM)	**ORF1p + 100 nM RNA** Average peak position (± SEM)
3-mer	104.1 ± 5.4	123.0 ± 6.2	130.1 ± 4.7
6-mer	231.8 ± 12.9	246.7 ± 6.9	n.a.
9-mer	330.7 ± 17.4	379.8 ± 9.7	n.a.
12-mer	448.32 ± 18.4	n.a.	n.a.

Table S3. Average peak position in histograms of mass photometry measurements of ORF1p-WT with different RNA length. N=3.

**RNA length**	**Average peak position of 1 trimer on RNA (± SEM)**	**Average peak position of 2 trimers on RNA (± SEM)**
30 nt	130.4 ± 3.7	n.a.
60 nt	164.5 ± 2.2	n.a.
70 nt	138.5 ± 2.4	n.a.
80 nt	142.0 ± 1.0	n.a.
90 nt	144.8 ± 3.6	294.2 ± 8.2
100 nt	146.3 ± 3.9	302.0 ± 6.0
110 nt	150.6 ± 4.5	310.9 ± 7.5
120 nt	154.7 ± 1.8	323.1 ± 3.6

Table S4. Average peak position in histograms of mass photometry measurements of ORF1p variants. WT-ORF1p: N=6; K3A/K4A-ORF1p: N=5; ΔStammer-ORF1p: N=4; His-TEV-ORF1p: N=5.

	**WT-ORF1p** Average peak position (± SEM)	**K3A/K4A-ORF1p** Average peak position (± SEM)	**ΔStammer-ORF1p** Average peak position (± SEM)	**His-TEV-ORF1p** Average peak position (± SEM)
1-mer	45.8 ± 5.8	n.a.	61.8 ± 7.6	53.8 ± 3.6
3-mer	106.49 ± 5.2	112.4 ± 7.3	109.3 ± 7.0	116.8 ± 2.8
6-mer	234.9 ± 11.5	244.7 ± 4.9	230.8 ± 22.0	259.8 ± 14.5
9-mer	352.5 ± 18.5	366.8 ± 8.6	339.9 ± 38.2	319.5 ± 17.4
12-mer	477.5 ± 21.8	486.6 ± 13.0	452.3 ± 45.4	n.a.

Data S1. Excel file containing individual data values, means, N and p values sorted by figure.
